# P014 Tibial fractures in patients on methotrexate—think about methotrexate osteopathy

**DOI:** 10.1093/rap/rkag113.044

**Published:** 2026-11-03

**Authors:** Hlaing Myat Chit Su, Jessica Chan, Jennifer Hannah, Sian Griffith

**Affiliations:** East Surrey Hospital, Redhill, United Kingdom; East Surrey Hospital, Redhill, United Kingdom; St George’s Hospital, London, United Kingdom; East Surrey Hospital, Redhill, United Kingdom

## Abstract

**Introduction:**

Methotrexate (MTX) osteopathy is a triad of bone pain, osteoporosis and atypical atraumatic stress fractures of the lower limbs. The specific radiological finding (a band-like/ meander shape running along the epimetaphysis) differs from osteoporotic fractures. Diagnosis relies on MRI which shows early stress fractures and osteolytic change. It is an important diagnosis as MTX is so widely used in rheumatological practice. Here, we discuss a patient who was on long term MTX and developed MTX osteopathy.

**Case description:**

A 61-year-old female with axial spondyloarthropathy with peripheral joint involvement diagnosed in 2002 presented in July 2025, with a 10-month history of bilateral ankle and midfoot pain. The pain sounded inflammatory in nature,was severe enough to necessitate a change to a sedentary job and disturbed her sleep. Examination demonstrated preserved range of movement in both ankles, with significant pes planus and no tendon tenderness. Ultrasound scan showed large effusion in the ankle joint but the severity of symptoms was disproportionate prompting MRI of both ankles.

MRI revealed a left distal tibia insufficiency fracture, a right distal tibia incomplete transverse fracture and calcaneal insufficiency fractures. Radiology MDT concluded these findings were characteristic of Methotrexate(MTX) osteopathy, with meandering epi metaphyseal changes in the distal tibia in the left ankle and calcaneus and lateral process of talus.

Previous treatment included Sulfasalazine (2003-2015) and Methotrexate (max dose 20mg weekly) (2015-present). She had multiple intra articular steroid injections (knees) but no oral steroids.

She received alendronate (2018-2021) (for low measured bone mass but no previous fractures.). In 2023, she had a fall from standing height resulting in an olecranon fracture and subdural haematoma (treated with burr hole evacuation, with no neurological sequelae). DEXA (2023) showed lumbar spine (LS)T-score -3.9, femoral neck (FN)T-score -1.5. Procollagen Type 1 N-Terminal Propeptide (P1NP) was low (23 ug/L). Alendronate was restarted that time.

She has no major risk factors for osteoporosis. DEXA (2025) showed LS T- score -3.9, FN T-score -1.3. Routine bloods, and osteoporosis screening bloods were normal.FRAX plus score is 13% risk of major osteoporotic fracture in the next 10 years and a 2.2% of hip fracture.

She was treated conservatively with walking boots and walking aids. Methotrexate was stopped, alendronate was switched to teriparatide, and she made a full recovery.

**Discussion:**

MTX is an anti-metabolite commonly used as first line disease modifying agent for many immune mediated conditions. It is associated with various potential side effects, and MTX osteopathy is a rare side effect related to long term use. MTX osteopathy was first described in 1970 in children receiving high dose MTX for acute lymphoblastic leukemia. However, 45% of cases occur in patients on low to moderate doses. Around 50% of patients have not had systemic steroids for more than 3 years, suggesting an independent effect of MTX. Around a half of the patients with MTX osteopathy meet densitometric criteria for osteoporosis. However, fractures due to methotrexate osteopathy tend to affect the lower extremities—in order of frequency: distal tibia, calcaneus and proximal tibia. Over half of cases have bilateral fractures, and in one quarter they are recurrent.

The pathognomonic MRI finding is a band- or meander-shaped appearance along the growth plate, referred to as epimetaphyseal osteolysis or band-like sclerosis. The mechanism and prevalence of MTX osteopathy are not clear. Postulated mechanisms are that methotrexate impairs osteoblastic cell function via upregulation of Notch2 signalling and attenuation of Wnt/beta-catenin signaling. It may also promote osteoclastogenesis via increased TNF-alpha, IL-1 and IL-6. Osteoporosis is a risk factor for methotrexate osteopathy. Cessation of MTX is the key management. Some patients have also been treated withs standard osteoporosis treatments such as alendronate, zoledronate, denosumab or teriparatide.

**Key learning points:**

• Rheumatologists need to be aware of MTX osteopathy because the symptoms can be confused with a flare of inflammatory arthritis. It causes atypical stress fractures predominantly in the lower extremities, particularly across the growth plates of the distal tibiae. Patients may need imaging with MRI for diagnosis.

• Almost half of the MTX osteopathy patients have both MTX osteopathy and osteoporosis. It is important to stop methotrexate in all patients. There is not enough evidence to give definitive advice on whether patients with low bone mass (with or without typical osteoporotic fractures) also need treatment for osteoporosis and it is not clear whether these cases need to be managed differently.

